# Supplementary material for: Microbial Diversity Associated with Gwell, a Traditional French Mesophilic Fermented Milk Inoculated with a Natural Starter
Source: Microorganisms. 2020 Jun 30;8(7):982. doi: 10.3390/microorganisms8070982 (PMC7409170; doi:10.3390/microorganisms8070982)
Supplement: Supplementary file 1 [file microorganisms-08-00982-s001.zip › microorganisms-840804-Supplementary.edited/Supplementary_tables.edited.pdf]

Table S1: Detailed list of the primers used for the metabarcoding analysis. Illumina tails are showed in bold and frame-shift spacers are underlined.

Genomic region	Primer name	Primer sequence
16S V3-V4	Illu4_16SV3F	**TCGTCGGCAGCGTCAGATGTGTATAAGAGACAG**NNNNTACGGRAGGCAGCAG
16S V3-V4	Illu6_16SV3F	**TCGTCGGCAGCGTCAGATGTGTATAAGAGACAG**NNNNDVTACGGRAGGCAGCAG
16S V3-V4	Illu8_16SV3F	**TCGTCGGCAGCGTCAGATGTGTATAAGAGACAG**NNNNNNRMTACGGRAGGCAGCAG
16S V3-V4	Illu4_16SV4R	**GTCTCGTGGGCTCGGAGATGTGTATAAGAGACAG**NNNNTACCAGGGTATCTAATCCT
16S V3-V4	Illu5_16SV4R	**GTCTCGTGGGCTCGGAGATGTGTATAAGAGACAG**NNNNHTACCAGGGTATCTAATCCT
16S V3-V4	Illu6_16SV4R	**GTCTCGTGGGCTCGGAGATGTGTATAAGAGACAG**NNNNNWTACCAGGGTATCTAATCCT
ITS2	Illu4_ITS3tagmix1	**TCGTCGGCAGCGTCAGATGTGTATAAGAGACAG**NNNNCTAGACTCGTCATCGATGAAGAACGCAG
ITS2	Illu6_ITS3tagmix1	**TCGTCGGCAGCGTCAGATGTGTATAAGAGACAG**NNNNDVCTAGACTCGTCATCGATGAAGAACGCAG
ITS2	Illu8_ITS3tagmix1	**TCGTCGGCAGCGTCAGATGTGTATAAGAGACAG**NNNNNNRMCTAGACTCGTCATCGATGAAGAACGCAG
ITS2	Illu4_ITS4ngs	**GTCTCGTGGGCTCGGAGATGTGTATAAGAGACAG**NNNNTTCCTSCGCTTATTGATATGC
ITS2	Illu5_ITS4ngs	**GTCTCGTGGGCTCGGAGATGTGTATAAGAGACAG**NNNNHTTCCTSCGCTTATTGATATGC
ITS2	Illu6_ITS4ngs	**GTCTCGTGGGCTCGGAGATGTGTATAAGAGACAG**NNNNNWTTCCTSCGCTTATTGATATGC

Table S2: Number of OTUs for the different species identified in the metabarcoding analysis. Total number of OTUs found across all samples, mean and median of the number of OTU per sample are shown.

Species	total number of OTU	mean number of OTU per sample	median number of OTU per sample
Enterococcus sp.	1	1.00	1.00
Lactobacillus acidophilus	2	2.00	2.00
Lactobacillus helveticus	1	1.00	1.00
Lactococcus lactis	5	2.31	2.00
Lactococcus sp.	3	1.20	1.00
Streptococcus salivarius	1	1.00	1.00
Streptococcus saliviloxodontae	2	1.44	1.00
Streptococcus sp.	2	1.17	1.00
Geotrichum candidum	1	1.00	1.00
Kazachstania servazzii	1	1.00	1.00
Saccharomyces cerevisiae	1	1.00	1.00
Saccharomycetales sp.	2	2.00	2.00
Yarrowia lipolytica	1	1.00	1.00
